# The human spinothalamic tract: lessons from cordotomy

**DOI:** 10.1093/braincomms/fcaf237

**Published:** 2025-06-17

**Authors:** Anthony K Allam, Michael Benjamin Larkin, Ishan A Patel, Mohammed Hasen, David Mears, Patrick Dougherty, Ashwin Viswanathan

**Affiliations:** Department of Neurosurgery, Baylor College of Medicine, Houston, TX 77030, USA; Department of Neurosurgery, Baylor College of Medicine, Houston, TX 77030, USA; Department of Neurosurgery, Baylor College of Medicine, Houston, TX 77030, USA; Department of Neurosurgery, Imam Abdulrahman bin Faisal University, Dammam 34212, Saudi Arabia; Department of Anatomy, Physiology & Genetics, USUHS School of Medicine, Bethesda, MD 20814, USA; Department of Pain Medicine-Research, The University of Texas MD Anderson Cancer Center, Houston, TX 77030, USA; Department of Neurosurgery, Baylor College of Medicine, Houston, TX 77030, USA

**Keywords:** spinothalamic tract, STT, cordotomy, complications, CT-guided

## Abstract

The spinothalamic tract has long been known as a primary conductor of nociceptive information, with its anatomical and physiological understanding evolving over centuries of research. This comprehensive review traces the history of the spinothalamic tract beginning with Brown-Sequard’s 1860 report of contralateral analgesia following a hemisection of the spinal cord. As clinical and surgical interventions, such as cordotomies have advanced, so did our understanding of the spinothalamic tract’s function and anatomy. The spinothalamic tract’s role as a crossed pathway conducting pain and temperature sensations was solidified by the mid-20th century. However, intricate details of its somatotopic arrangement, anatomical distributions and sensory modalities were further refined by subsequent studies. Additionally, by examining complications of cordotomies, such as Ondine’s Curse (central hypoventilation syndrome), urinary and autonomic dysfunction, motor weakness and late-onset new pain, the intricate relationships between the spinothalamic tract, the ascending and descending reticular tracts, the spinocerebellar tracts and other vital pathways governing micturition and autonomic functions were discovered.

## Teaser

Allam *et al*. report on the somatotopic and functional organization of the spinothalamic tract, drawing on insights from animal and human studies involving cordotomy-induced lesioning. Furthermore, by analyzing cordotomy complications, they elucidate the relationship between the spinothalamic tract and adjacent spinal pathways.

## Graphical Abstract

**Graphical Abstract:**
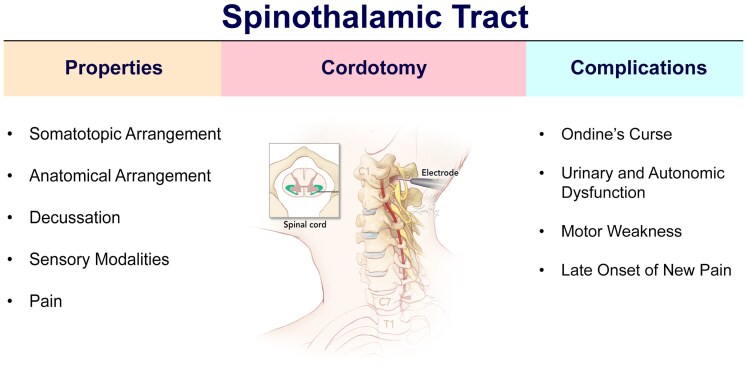

## Introduction

The existence of a spinal tract that conducts nociceptive information has long been known, with the first formal description arising from a case report in 1860.^1^ Brown-Sequard reported contralateral analgesia and thermoanaesthesia following a hemisection of the spinal cord.^1^ Gower in 1886 further characterized this tract as a crossed pathway in the anterolateral quadrant.^2^ Shortly after that, Spiller discovered a patient with bilateral tuberculomas of the anterolateral quadrant who experienced complete analgesia and thermoanaesthesia caudal to the lesion.^3^ Based on this finding, Spiller convinced Martin to perform the first cordotomy in 1912 with a marked reduction in pain and temperature sensations.^4^

Early cordotomies for pain were frequently performed using an open procedure where the surgeon would expose the spinal cord and divide the anterolateral quadrant. However, in the 1960s and 1970s, the percutaneous approach became popularized and was frequently used until the broader adoption of opioids and other neuromodulatory techniques replaced ablative procedures.^5^ In the past couple of decades, there has been renewed interest in CT-guided cordotomies. Several recent studies, including a clinical trial, have supported its safety and effectiveness in reducing unilateral opioid-resistant cancer-related pain.^6-8^

**Figure 1:**
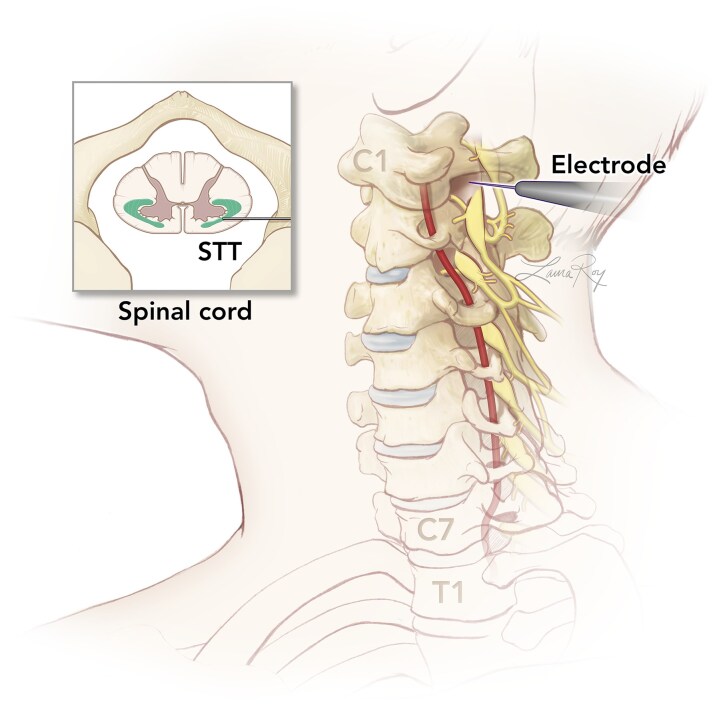
**Depiction of a CT-guided cordotomy.** STT, spinothalamic tract; CT, computed tomography. Figure created by Laura Roy.

CT-guided cordotomies are performed percutaneously through a lateral approach to the C1–C2 interspace in humans (Figure 1). Using real-time intraoperative CT, a 20G radiofrequency electrode is advanced into the anterolateral quadrant of the spinal cord in the awake patient. Test stimulation is used to confirm physiologically that the electrode is within the spinothalamic tract (STT) and to ensure that corticospinal fibres are a safe distance from the electrode.^9^ Radiofrequency lesioning is then performed to interrupt the ascending fibres of the STT. CT-guided cordotomy has significantly advanced the field by dramatically reducing complication rates and enhancing procedural efficacy.

Human and animal studies of cordotomy performed over the past 100 years have provided significant insight into the anatomy and function of the STT. This report will review the knowledge gained from basic science and human cordotomy studies.

## Materials and methods

PubMed was used as the primary database for electronic article searching. In addition, some studies were identified using citation searching. The formal search consisted of the following structures: ‘(cordotomy AND spinothalamic)’ and ‘(cordotomy AND pain).’ Only publications in English that were retrievable were considered.

### Animal studies

The final papers were chosen based on the following criteria: (i) the study targeted the STT, (ii) the study contained data from monkeys and (iii) written in English. Exclusion criteria included the exclusive presence of animals other than monkeys.

### Human studies

The final papers were chosen based on the following criteria: (i) the patients had a cordotomy performed, (ii) the study contained formal psychophysical, imaging, or histological data studying the effects of the cordotomy and (iii) the study was written in English. Exclusion criteria included reports with only electrophysiological or clinical outcome data.

## Results

**Figure 2:**
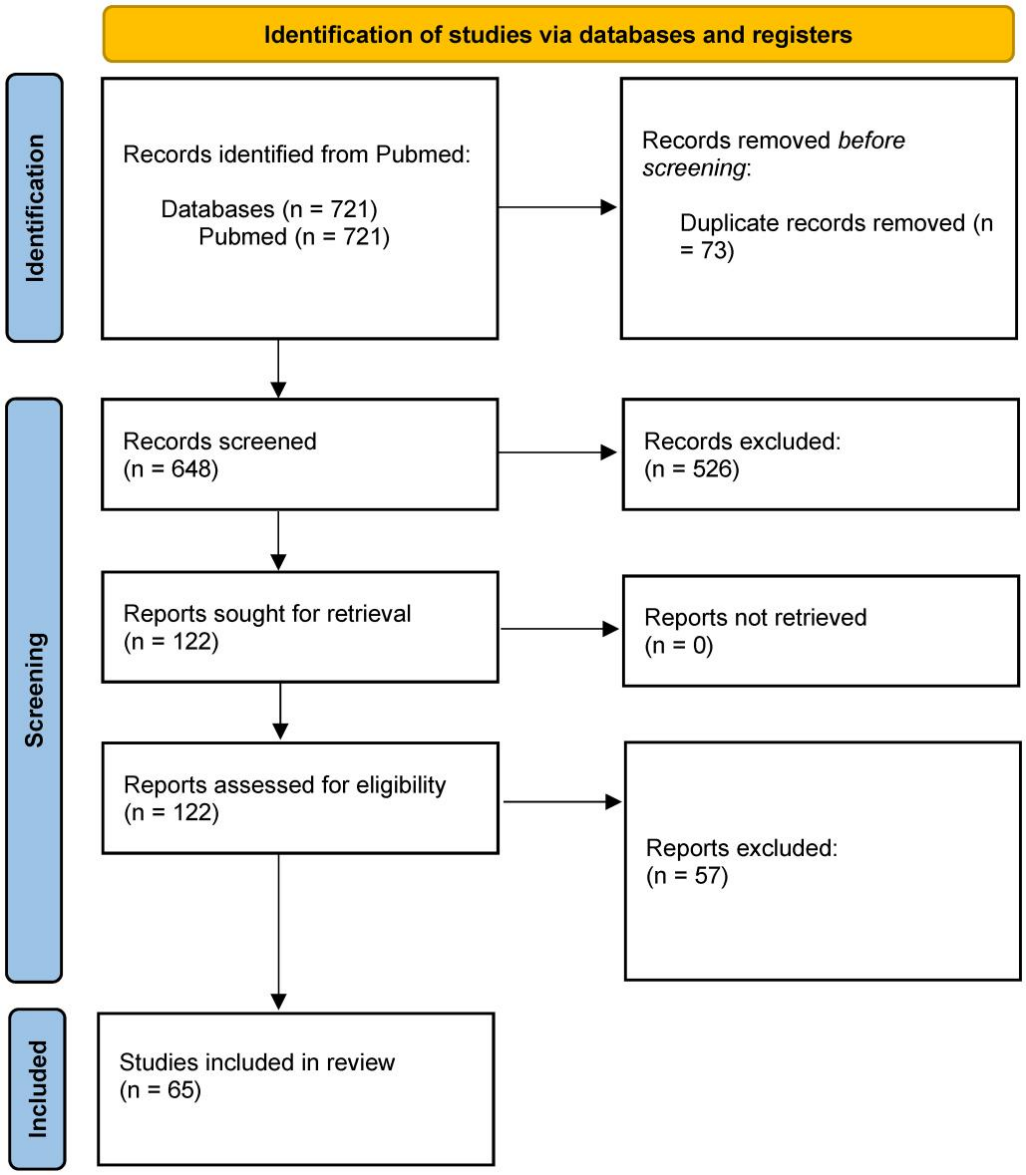
**PRISMA method outline.** PRISMA, Preferred Reporting Items for Systematic Reviews and Meta-Analyses.

The original search was performed on 6 September 2022. This search yielded a total of 723 results. These results were then screened through an abstract and title review before undergoing a full-text review (Figure 2). A total of 22 animal papers fit our search’s inclusion and exclusion criteria (Supplementary Table 1). A total of 45 human papers fit our search’s inclusion and exclusion criteria (Supplementary Table 2).

## Discussion

Although the first cordotomy was performed in 1912, it was not until years later that Otfrid Foerster investigated the anatomical basis of pain relief.^10,11^ Early investigators used clinical, anatomical and histological correlations to understand the arrangement, pathways and sensory modalities carried by the STT.^10-14^ By the 1950s, there was a consensus that the STT was a crossed pathway that decussated within two levels of entering the spinal cord and carried the sensations of pain, temperature and itch. Even during this time period, it was known that the STT had a somatotopic arrangement, with lumbar fibres being located dorsolaterally and cervical fibres being located ventromedially. Furthermore, through clinical evidence, several complications related to cordotomy were discovered: pulmonary dysfunction, bladder dysfunction, autonomic dysfunction, motor weakness and late onset of new pain. Several critical studies since then have further characterized the STT and contributed to our understanding of cordotomies.

### Morphology and physiology of the STT

#### Somatotopic arrangement

Studies by Roth *et al*., White *et al*., Lahuerta *et al*., Vedantam *et al*. and Honey *et al*. have since validated earlier studies indicating that the STT had a general somatotopic arrangement with fibres from the lower body being located dorsolaterally and fibres from the upper body being located ventromedially.^15-19^ However, White *et al*. noted that scattered fibres from the leg and buttocks can be found more ventrally in the STT, and Honey *et al*. noted that STT fibres from the arm could be seen more dorsomedially than previously thought.^16,19^ Despite the slight variabilities in somatotopic arrangement, electrode testing is used to ensure the ablation of the desired fibres.

#### Anatomical arrangement

Alongside the discovery of somatotopic arrangement, the physical extent of the STT in the spinal cord has also been elucidated. White *et al*. remarked that in the thoracic cord, sacral fibres were located dorsally just in front of the dentate ligament and laterally just deep to the ventral spinocerebellar tract.^16^ Building on this study at the cervical level, Lahuerta *et al*. illustrated that nociceptive fibres at the C2 level were located anterior to the dentate ligament and extended to a line drawn perpendicularly to the medial angle of the ventral horn.^17^ However, there have been case reports where nociceptive fibres, presumably of the STT, have been found in the posterolateral quadrant.^20^ In 1989, Apkarian published a study demonstrating the presence of a dorsolateral STT in monkeys.^21^ He noted that lesioning of the ventral cord in the thoracic spine led to incomplete pain relief.^21^ This result was corroborated by Zhang *et al.* who demonstrated that thoracic and lumbar fibres of the STT reside dorsal to the dentate ligament.^22^ However, the fibres of the STT shift ventrally as they ascend in the spinal cord and are exclusively located ventral to the dentate ligament at the cervical level.^22^ This lends support to the hypothesis that an open thoracic cordotomy performed in humans may lead to incomplete pain relief to due preservation of STT fibres located dorsal to the dentate ligament. However, with the high cervical percutaneous approach, the interventionalist has access to all the spinothalamic fibres below the C5 dermatome, and hence more complete pain relief is possible.

#### Decussation

A multitude of studies have shown that the STT decussates within two segments of entering the spinal cord based on lesions at the cervical level; however, in a series of studies, White *et al*. concluded that at the thoracic level, the decussation of the STT can vary between individuals ranging from one to six segments.^16^ Although there was controversy during the 1950s about whether the STT decussated diagonally or transversely, recent studies have shown that the STT does indeed decussate transversely through the anterior white commissure.^23^ However, it is essential to note that there is a spectrum of anatomical variation ranging from uncrossed to posteriorly located to dysfunctional spinothalamic tracts.^20,24-28^

#### Sensory modalities

Early reports of cordotomies suggested that the sensations of nociception and temperature ran together and were inseparable; however, several case reports, animal studies and human studies have since repudiated this view.^16,18,25,29-33^ It is now known that while closely related, nociception and temperature are separate entities that can be abolished independently of each other. Friehs *et al*. in 1995 and Vedantam *et al*. in 2018 proposed similar but unique distributions for nociceptive and temperature fibres.^18,30^ Vedantam *et al*. hypothesized that the fibres mediating temperature are located dorsally in a distinct region compared to the fibres mediating sharp pain, albeit in a similar somatotopic arrangement.^18^ In contrast, Friehs *et al*. hypothesized that fibres relating to temperature are located behind the fibres mediating sharp pain of each respective level (e.g. cervical, thoracic, etc.) rather than in a distinct region.^30^ Along with nociception and temperature, it has been shown that the STT carries crude touch and pressure. In a unique case study of patients with a combined commissural myelotomy and anterolateral cordotomy, Nathan *et al*. surmised that the dorsal columns and the STT carry redundant information regarding tactile and pressure sensibility.^34^ It is now known that the human STT does transmit information about pressure and crude touch, albeit more ventromedially than nociception and temperature. However, as mentioned above, an anterolateral cordotomy does not produce deficits related to tactile, pressure, proprioceptive or vibratory sense, as the dorsal columns carry enough information to avoid a clinical deficit.^31,35-37^ Nevertheless, recent research has shown that information regarding crude touch in the STT is likely essential for experiencing referred pain.^38,39^ Furthermore, there has been theories that while the physical sensation of touch is carried through the dorsal columns, the affective-emotional aspect can be carried by C-tactile fibres in the STT. A study by Marshall *et al.*, disproved this theory and instead attributed the affective-emotional aspect of touch to hedonic and discriminative spinal processing.^40^ Finally, it has been known that the STT carries information regarding itch, as the sensation of itch is likewise abolished after a cordotomy.^31,37,41^ There has been debate about whether the sensation of itch is carried in separate fibres similar to nociception and temperature or is a result of higher-order processing. There is evidence in animals that neurons that respond exclusively to itch do exist; however, no such correlate has been found in humans yet.^42^ Currently, there is no evidence to suggest that neurons that respond exclusively to itch exist in humans, but instead, the itch sensation is carried by neurons that respond to itch and nociception.

#### Pain

**Figure 3:**
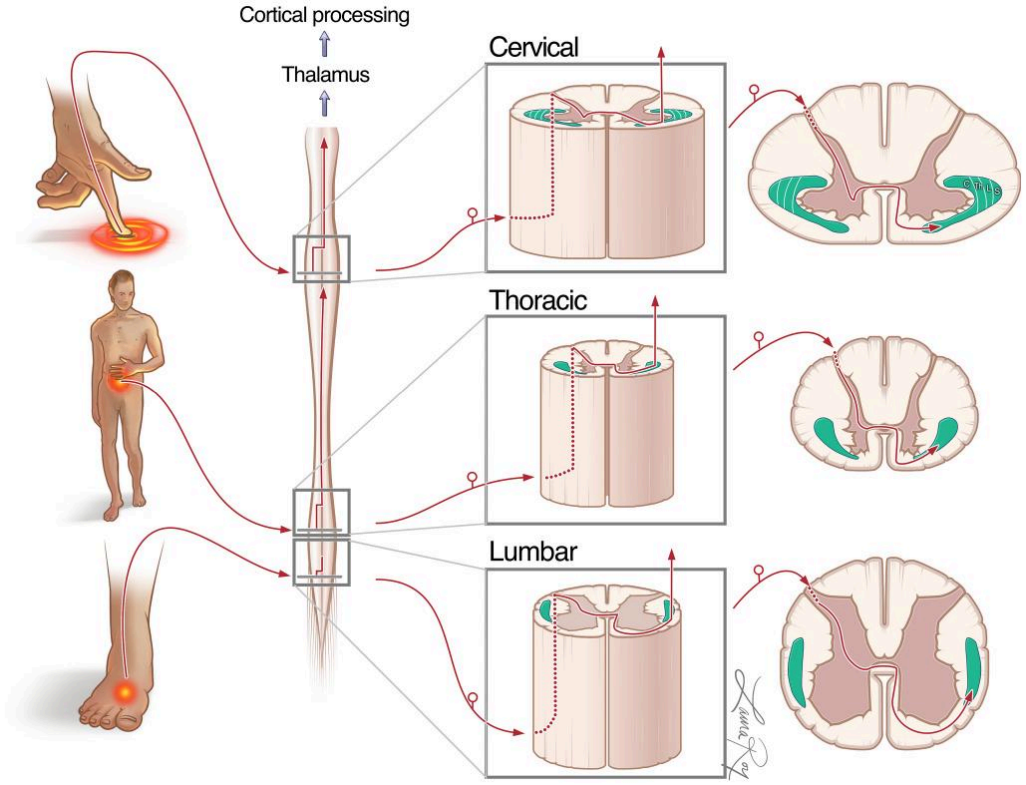
**Depiction of the spinothalamic tract in the human spinal cord.** Anatomic and functional arrangement of the spinothalamic tract is depicted at cervical, thoracic, and lumbar levels. C, cervical; Th, thoracic; L, lumbar; S, sacral. Figure created by Laura Roy.

Somatic pain carried by the STT is transmitted via three major classes of nociceptors: thermal, mechanical and polymodal. The first two categories consist of myelinated and thinly myelinated Aδ fibres, while polymodal nociceptors consist of non-myelinated C-fibres.^43,44^ When entering the dorsal tract, these fibres can ascend cranially through Lissauer’s tract before synapsing on various areas of the Rexed laminae. Several animal studies have shown that fibres of the STT primarily synapse on Laminae I and Laminae V (deep dorsal horn) with smaller contributions from Laminae VI to VIII.^21,44-49^ In humans, it has been shown that C-fibres first synapse on Laminae I before projecting to the VMPo and Brodmann area 3a of the somatosensory (S1) cortex; however, the importance of this pathway in nociception is debated.^50^ In contrast, Aδ fibres primarily synapse on both Laminae I and V before projecting to the VPL and Brodmann area 3b/1 of the S1 cortex.^50^ Distinct neurons (i.e. nociceptive specific, cold cell and polymodal nociceptive) mediating aspects of nociception and temperature have been shown to synapse on Laminae I.^29^ Electrophysiological studies of anterolateral quadrant neurons in humans and animals demonstrate that, although concurrent activation of Laminae I and V can signal pain, activation of Lamina V alone is sufficient to evoke a pain response.^51,52^ Thus, Laminae I and V may each contribute significantly to the analgesic and thermoanaesthetic effects of cordotomy, and further research is required to understand their interplay. In a series of studies, Craig *et al*. illustrated that the primate STT is composed of anatomically and functionally differentiable components as he was able to track varying laminar components of the STT to distinct thalamic subregions.^53-58^ Although the presence of a dorsal STT in the dorsal funiculus carrying primarily Laminae I neurons has been proven in monkeys; there has been no such correlation found in humans^21,47,48,59^ (Fig. 3**)**.

### Complications of a cordotomy

#### Ondine’s curse

Many neuroanatomical discoveries have been made due to early complications from cordotomy. One such and a rather peculiar complication was Ondine’s curse: central hypoventilation syndrome (CHS). This complication was initially noticed after a bilateral high cervical cordotomy was performed.^13,60-64^ Since then, numerous studies have cautioned that (i) CHS may result following a unilateral high cervical cordotomy if pre-existing pulmonary pathologies are present, (ii) a bilateral cervical cordotomy, having pre-existing pulmonary pathology, or achieving deeper lesions significantly increase the risk of developing CHS and (iii) the effects of the above factors are additive.^60-64^ A number of studies have looked at specific pulmonary pathologies that predispose individuals to CHS, but none have been definitely found.^61,63,65-68^ However, a more recent study has suggested that even in the presence of pulmonary pathology, a unilateral cervical cordotomy may still be performed without significant effect on respiratory function.^65^ Early researchers surmised that CHS resulted from the disruption of descending motor fibres within the corticospinal tract. However, many patients could still breathe without difficulty throughout the day and had no evidence of corresponding motor deficits.^69,70^ As a result, the focus was shifted to what is now known as the reticulospinal tract, which is responsible for several autonomic functions such as breathing, heart rate, circulation, etc.^70^ In a study by Hitchcock *et al*. in 1967, the authors postulated that the posterior portions of the respiratory tract (i.e. intercostal muscles) were associated with posterior portions of the STT but that the area that had the most effect on the respiratory system (i.e. diaphragm) was located closer to the cervical segments of the STT.^62^ These findings supported their hypothesis that higher levels of analgesia following a cervical cordotomy (i.e. deeper lesions) increase the risk of damaging the reticulospinal tract and thus developing CHS. Furthermore, the risk is increased following a bilateral cordotomy, indicating that one intact reticulospinal tract is sufficient to mediate normal respiration. However, a pre-existing pulmonary pathology could potentially render the affected lung insufficient to maintain adequate ventilatory activity after a unilateral loss of lung function produced by a cordotomy. Although the risk of central hypoventilation is lower with the use of CT-guided cordotomy, caution remains advised, but ultimately the patient’s therapeutic goals, in conjunction with in-depth patient counselling on the risks and benefits, should guide clinical decision-making for proceeding with cordotomy.

#### Urinary and autonomic dysfunction

Other complications noticed after cordotomy were urinary and other autonomic dysfunctions (i.e. Horner’s syndrome, orthostatic hypotension, anhidrosis, etc.).^15,17,31,41,71-74^ From the earliest cordotomies, it was known that a unilateral cordotomy could result in transient urinary incontinence, while a bilateral cordotomy more commonly resulted in permanent dysfunction. Early researchers felt that the bladder was bilaterally innervated, needing only unilateral innervation to function normally. Hitchcock *et al*. hypothesized that the vesicomotor pathway lay within 3 mm of the cord surface in the region of the dentate ligament explaining why bilateral cordotomies that produced sacral analgesia, as a result of lesions near the dentate ligament, frequently resulted in micturition disorders.^72-74^ Along with bladder dysfunction, sympathetic disruption leading to Horner’s syndrome was also commonly noticed after cordotomies. There was much debate at the time regarding the location of autonomic fibres within the spinal cord. Some felt the fibres ran in the anterolateral quadrant, while others hypothesized they were closer to the pyramidal tracts.^13,14,75,76^ Interestingly, however, Roth *et al*. discovered that the autonomic pathways within the spinal cord were instead widely distributed in the anterolateral quadrant and that a significant portion of these fibres was not directly related to the corticospinal tract.^15,71^ Supporting this discovery, Verlato *et al*. noticed that after a unilateral cordotomy autonomic fibres were disrupted as evidenced by changes in heart rate variability.^77^ Furthermore, Johnson *et al*. noted that vasomotor and sweating functions were affected in a more dissociated manner, indicating that different aspects of the autonomic system are within various portions of the spinal tract.^71^ It is now known that the reticulospinal tract carries autonomic information and that the tract is widely distributed across the anterolateral quadrant in humans.^78^ Additionally, it is worth noting that autonomic dysfunction might also arise from the disruption of STT collaterals extending into the midbrain, pons and medulla, which mediate these functions through various tracts, such as the spino-mesencephalic tract and the spinoreticular tract. All of these tracts include large populations of dual or even triple projecting STT axons.

#### Motor weakness

Motor weakness following a cordotomy can be particularly debilitating but is not frequently encountered today due to the use of intraoperative CT scans. Early researchers focused on localizing motor pathways. Nathan *et al*. reported that incisions within the ventral half of the spinal cord could be made without causing any motor disturbances.^79^ The more posterior the incision reached, the greater the motor deficits.^79^ Furthermore, the authors noticed that the corticospinal tract was more posteriorly located in the cervical regions than the spinal cord’s lower areas.^79^ As such, conducting a cordotomy in the cervical region and limiting the lesion to the ventral portion of the spinal cord anterior to the dentate ligament can reduce the risk of motor complications. In an interesting study, Nathan *et al*. found that patients can partially recover motor function if the contralateral corticospinal tract is intact.^80^ Therefore, it is crucial to be extra cautious of the corticospinal tracts when performing a bilateral cordotomy.

#### Late onset of new pain

Finally, one of the most diametric complications arising from an anterolateral cordotomy is the late onset of new pain in the form of dysesthesias, mirror pain syndrome, or referred pain.^81-83^ It is estimated that anywhere from 33% to 73% of patients can experience mirror pain following a unilateral cordotomy.^82-85^ Furthermore, it is known that the initial benefits of cordotomy wain over a couple of years. This then begs the question, how does pain return if the primary tract of nociception has been disrupted? The answer to this question may result from spinal cord plasticity from uncrossed fibres of the STT, the spinoreticular tract, multisynaptic ascending pathways and even the dorsal columns. Animal studies have shown that spinoreticular neurons from Laminae VII have large bilateral receptive fields that run in both crossed and uncrossed pathways.^86,87^ Furthermore, these neurons have large inhibitory receptive fields which if damaged by a cordotomy can result in increased transmission of nociceptive signals due to decreased inhibition of neighbouring neurons.^86^ Another possible explanation not often mentioned is the presence of multisynaptic pathways consisting of short neurons that are capable of transmitting nociceptive pain.^88,89^ These pathways may bypass the lesion within the STT entirely and instead engage circuits that are both ipsilateral and contralateral to the lesion, as mentioned above, resulting in the formation of new mirror pain.^88,89^ Additionally, dorsal columns carry visceral nociceptive information (dorsal visceral pain pathway), and following a cordotomy, these afferent pathways may also become involved in transmitting somatic information.^90,91^ Although nociception is often considered a hard-wired pathway, there is evidence that pain is the summation of various inhibitory and excitatory effects of many central sensory pathways.^92,93^ Brown-Sequard, right before he died, published a study noting that a second hemisection performed caudal to the first one could reverse the analgesic effects of the first lesion.^94^ Denny-Brown provided further support for Sequard’s new view by conducting double hemisection studies and noting that the second lesion abolished the analgesia produced by the first hemisection.^95^ He hypothesized that the second hemisection disrupted descending inhibitory controls engaged by the first hemisection. This view was further championed by Bowsher *et al*., who speculated that mirror pain occurred due to the destruction of tonic descending inhibitory pathways, which generally suppress the ipsilateral receptive field of nociceptive neurons with bilateral symmetrical receptive fields.^81^ While this theory explained mirror pain, it was inadequate in explaining referred pain that extends both cranially and caudally to the lesion. Nagaro *et al*. in 1993 proposed that in normal conditions, the STT exerts negative feedback on subsidiary pain pathways preventing impulses from travelling in those circuits.^84^ However, once feedback inhibition is removed after a cordotomy, the subsidiary pathways can then propagate signals.^84^ Finally, a theory proposed by Treister *et al.* in 2022, attributes mirror pain syndrome to central sensitization.^82^ The authors found that pre-operative temporal summation values, an indicator of central sensitization, was able to predict the occurrence of post-operative mirror pain syndrome.^82^ While the entirety of the neural circuitry of the pain pathways within the spinal cord remains to be discovered, it is important to recognize that current evidence tells us that somatic nociception involves more than just the spinothalamic tracts.

### Radiographic analysis

Researchers have also delved into the supra-spinal effects of cordotomy and have attempted to predict cordotomy outcomes utilizing radiographic imaging and machine learning algorithms. Piero *et al*., with the use of positron emission tomography, found that patients with chronic pain had decreased synaptic activity within the dorsal anterior quadrant of the contralateral thalamus.^96^ Furthermore, he noticed that after a cordotomy, activity in the area returned to normal indicating that a cordotomy not only affects the STT but also supra-spinal circuits that may be responsible for maintenance of chronic pain.^96^ These supra-spinal circuits are also pivotal in the development of other pain syndromes, such as thalamic pain syndrome. In this condition, damage to the ventroposterolateral and ventroposteromedial nuclei leads to profound central pain. Instead of merely disrupting the downstream spinothalamic and other nociceptive pathways, thalamic injury causes a loss of inhibitory control, resulting in amplified nociceptive signals transmitted from the thalamus to the cortex, where they are misinterpreted. More recent studies have aimed to characterize the effects of a cordotomy on the spinal cord utilizing advanced radiographic techniques. Gebarski *et al*. was one of the first researchers to use diffusion tensor imaging (DTI) to study the spinal cord after a cordotomy.^97^ He noticed that a cordotomy reduced the rostrocaudal directional DTI signal from the lesion in the spinal cord through the mesencephalon indicating that the analgesic effects of a cordotomy were due to focal ablation and the subsequent anterograde degeneration of the STT.^97^ Building on his work, Vedantam *et al*. noticed that a DTI scan could not only detect microstructural changes, but also predict early post-operative outcomes based on the mean diffusivity (MD) score.^98^ Further corroborating the predictive power of a DTI scan, Berger *et al*. noted that changes in MD and fractional anisotropy values represent tissue damage with an increased MD value linked to better clinical response.^85^ Additionally, the authors found that the size of the lesion did not affect outcomes once a minimum size was achieved.^85^ Translating the success of DTI scans in visualizing cordotomy outcomes to MRI scans, Vedantam *et al*. described the radiographic characteristics of a cordotomy lesion and in a later paper the authors were able to predict short-term cordotomy outcomes solely based on MRI features.^99,100^

### Limitations

This review article aims to summarize the collective knowledge regarding the pathways of somatic nociception that have been gained due to cordotomies in primate and human studies. While a considerable effort has been made to include all the salient points, many early articles were either not accessible or written in languages other than English. Albeit relevant information from a number of these articles are discussed within the cited papers included in this review, and their collective findings are summarized.

## Conclusion

Ever since the first cordotomy was performed in 1912, there has been a considerable amount of basic science and human research regarding the effects of STT lesioning. We now know that the STT is a crossed pathway that decussates within two segments of entering the spinal cord, at the cervical level, and carries the sensations of pain, temperature, itch, crude touch and pressure in a somatotopic arrangement. Although the STT is considered the primary nociceptive pathway, we now know that several other collateral pathways do exist and play a significant role in the transmission and disinhibition of nociceptive pathways. Furthermore, by analyzing the complications of cordotomies, we have learned a significant deal about the ascending and descending reticular tracts and the spinocerebellar tracts, along with the pathways that mediate micturition and autonomic functions. Hopefully, continued research will, one day, elucidate the exact mechanisms and nociceptive neural circuitry so that this procedure, and those like it, can provide the much sought-after long-term pain relief outcomes.

## Supplementary Material

fcaf237_Supplementary_Data

## Data Availability

Data sharing is not applicable to this article as no new data were created or analyzed in this study.
