# Supplementary material for: The human spinothalamic tract: lessons from cordotomy
Source: Brain Commun. 2025 Jun 17;7(3):fcaf237. doi: 10.1093/braincomms/fcaf237 (PMC12198438; doi:10.1093/braincomms/fcaf237)
Supplement: fcaf237_Supplementary_Data [file fcaf237_supplementary_data.pdf]

Supplementary Materials

Authors	Year	Methodology	Key Findings
**Albe-Fessard et al.** [1]	1973	Antidromic localization of STT neurons in 6 macaque monkeys after thalamic stimulation	A pathway having neurophysiological characteristics of layer V neurons project to the nucleus ventralis posterior and the adjacent VL. The afferent messages are conducted via Aδ and Aα fibers. The role of C fibers could not be analyzed.
**Kerr et al.** [2]	1975	Varying lesions of the ventral funiculus were made in 4 macaque monkeys. After 7-14 days, the spinal cord and brainstem were cut in the transverse plane and histologically stained.	The VSTT is an independent entity compared to the LSTT with analogous but not identical connections to the brain stem and thalamus. Furthermore, the VSTT has a smaller volume. The medial component of the VSTT is concerned with the affective motivational aspects of nociception while the lateral VSTT is related to discriminatory function.
**Trevino et al** [3]	1975	HRP was injected into the caudal diencephalon/ rostral mesencephalon of 5 cats and two rhesus monkeys. Retrograde transport was analyzed.	The STT in the lumbar region of monkeys is primarily composed of axons from laminae I, IV, and V, with some axons from VI, VII, and VIII.
**Willis et al** [4]	1979	HRP was injected into the thalamus of 12 macaque monkeys. Retrograde transport was analyzed at the cervical, thoracic, and lumbar levels.	Injections to the lateral thalamus labeled majority contralateral neurons from laminae I and V, while injections to the medial thalamus labeled majority contralateral neurons from laminae VI-VIII. In the conus medullaris, the STT cells were most labeled from laminae I, V, and VI.
**Vierck et al.** [5]	1979	Nine cebus monkeys were trained to escape electrical stimulation of either leg. Following varying lesions to the spinal cord, the monkeys were tested on the escape task.	The dorsal pathways do not play a major role in the conduction of information necessary for pain perception in monkeys. Furthermore, in monkeys, a bilateral lesion of the ventral columns is necessary for long-lasting decrease in pain.
**Levitt et al.**[6]	1983	Clinical observations of 6 macaque monkeys who underwent left unilateral thoracic cordotomy followed by right thoracic cordotomies in 4 monkeys	The release of deafferentation syndrome by a contralateral cordotomy is independent of the functional activity of the ipsilateral anterolateral tracts. The pathophysiological foci of chronic deafferentation syndrome is hypothesized to be at the level of the brain rather than the spinal cord.
**Greenspan et al.** [7]	1986	Four macaque monkeys were trained to escape electrocutaneous stimulation. Following a unilateral anterolateral cordotomy, the monkeys were then tested on the escape task	All monkeys had a large reduction in escape response along with longer latency to respond following contralateral stimulation. The monkeys reacted to low levels of stimulation, indicating that despite a higher detection threshold, a cordotomy

			did not eliminate sensibility for low levels of stimulation. Furthermore, cordotomies produced bilateral attenuation of reflexive amplitudes.
**Apkarian et al.[8]**	1989	WGA-HRP was injected into the thalamus of 3 macaque monkeys. Retrograde transport was analyzed at the lumbar level in intact spinal cords or in the presence of ipsilateral ventral or dorsolateral midthoracic spinal cord lesions.	A significant proportion of laminae I cell axons ascend to the thalamus in the contralateral dorsolateral funiculus. A few laminae I cell axons, and the majority of deeper laminae axons of the STT ascend in the contralateral ventral quadrant.
**Apkarian et al.[9]**	1989	WGA-HRP was injected into the thalamus of 11 macaque monkeys. Retrograde transport was analyzed at the lumbar level in the presence of ipsilateral ventral or dorsolateral midthoracic spinal cord lesions	In the macaque monkey, the DSTT is primarily composed of laminae I axons, while the VSTT is primarily composed of laminae IV-X axons. The DSTT was calculated to compose 25% of the total STT population.
**Apkarian et al. [10]**	1989	WGA-HRP was injected into the thalamus of 9 macaque monkeys. Anterograde transport was analyzed from the lumbar level in the presence of ipsilateral ventral or dorsolateral midthoracic spinal cord lesions	The termination of the DSTT and the VSTT largely overlap and innervate the lateral and medial thalamus. The authors hypothesized that the DSTT supplies the noxious spatial position while the VSTT supplies the noxious intensity channel.
**Vierck et al. [11]**	1990	Eighteen pig-tailed monkeys were analyzed on operant escape and reflex responses to electro-cutaneous stimulation before and after an anterolateral cordotomy. Histological correlations were also conducted.	The time course of recovery for operant and reflex responses differed. Operant responsivity to stimulation contralateral to the lesion varied between animals, with recovery associated with medially extensive lesions. Reflex magnitudes increased over months of testing, with greater recovery being associated with recovery of reactions to nociception.
**Ralston et al.[12]**	1991	Wheat germ agglutinin conjugated with horseradish peroxidase (WGA-HRP) was injected into the spinal cord of 6 macaque monkeys in conjunction with a contralateral cervical cordotomy. Anterograde transport was analyzed.	A substantial portion of the DSTT projects to the posterior nuclei of the caudal-ventral thalamus (Po/SG). Dorsolateral lesions up to the level of the denticulate ligament abolish WGA-HRP transport. Larger lesions that destroy the ALQ and most of the lateral column white matter except for the corticospinal tract also abolish WGA-HRP transport.
**Craig et al. [13]**	2000	Either injections of PHA-L, TRITC, or FITC were made in either the superficial cervical or lumbar dorsal horns in 10 macaque monkeys.	Lamina I axons were concentrated in the middle of the lateral funiculus. At the C1-2 level, these axons were more ventrally located, with a general topography arrangement consisting of superficially

		Anterograde transport was analyzed and correlated with histological sections.	located lumbar axons and medially located cervical axons. At the thoracic level, a few axons were found more dorsally and more ventrally.
**Weng et al.** [14]	2000	Neuronal recordings of the ventral posterior lateral nucleus of the thalamus in baseline and thoracic cordotomy groups were analyzed in response to formal sensory testing on six macaque monkeys. Histological correlations were made for the cordotomy group.	The study concluded that neurons responding to compressive and phasic brush stimuli but not brush-specific neurons showed increased spontaneous activity, increased responses evoked by cutaneous stimuli, and larger mean receptive field size in monkeys with a cordotomy compared to those with intact spinal cords. There was an increased frequency of spike-burst and non-burst events compared to the intact thalamus.
**Zhang et al** [15]	2000	Twenty-six lumbar STT cells were located using antidromic stimulation and traced through the thoracic and cervical spinal cord.	Axons of the STT shift ventrally and laterally as they ascend in the spinal cord. At the cervical spine level, all axons of the STT reside within the ALQ of the spinal cord. Axons from the SDH are dorsal to those from the DDH throughout the spinal cord.
**Craig et al.** [16]	2001	Seventeen lumbosacral STT neurons which were mechanically and thermally insensitive were identified by antidromic and orthodromic stimulation.	The study concluded that there exists lamina I STT neurons that lack sensitivity to mechanical or thermal stimuli but are rather histamine-selective and propagate the sensation of itch.
**Craig et al** [17]	2002	Lamina I STT neurons were double-labeled for calbindin and retrogradely transported WGA-HRP following injections to the thalamus. In addition, oblique transverse sections from upper cervical spinal segments were stained for calbindin-immunoreactivity.	Most laminae I STT cells of the primate are calbindin-immunoreactive and ascend in a bundle in the middle of the lateral funiculus in both monkeys and humans. The bundle has a variable location and extent in different individuals and may explain clinical findings
**Craig et al.** [18]	2004	Twelve lamina I neurons and 12 lamina V neurons were antidromically identified in 16 macaque monkeys. Electrophysiological recordings were obtained as various stimuli were tested.	Laminae I neurons, not laminae V neurons, are responsible for burning pain conducted by the STT.
**Craig et al.** [19]	2006	STT neurons from 11 macaque monkeys were retrogradely labeled with variously sized injections of CTb from the PL nucleus, specifically from the VMpo.	The study concluded that the VMpo nucleus receives input almost entirely from laminae I neurons in an anteroposterior topographic arrangement. The VMpo nucleus is a primate lamina I spinothalamocortical relay nucleus.
**Craig et al.** [20]	2006	STT neurons from 19 macaque monkeys were retrogradely labeled	The study concluded that the VPL nucleus receives STT input almost entirely from laminae V neurons,

		with variously sized injections of CTb from the VPL nucleus and VPI nucleus.	while the VPI nucleus receives STT input from both laminae I and V neurons. The VPL nucleus has a mediolateral topographic input, whereas the VPI nucleus has an anteroposterior topographic input.
**Craig et al. [21]**	2008	STT neurons from 14 macaque monkeys were retrogradely labeled with variously sized injections of CTb from the VL nucleus.	The study concluded that the VL nucleus receives input from laminae V and VII neurons that may be coextensive with its cerebellothalamic input. In addition, the VL nucleus is arranged in a mediolateral topographic fashion.
**Craig et al.[22]**	2014	Anterograde tracers were placed at different levels in VMpo based on the anteroposterior topographic organization of nociceptive units	The VMpo projection area in the dorsal posterior insular cortex provides the basis for the somatotopic representation of selectively nociceptive lamina I spinothalamic activity.

Supplementary Table 1: Summary of Animal Studies

Abbreviations: STT – Spinothalamic tract; VSTT – Ventral spinothalamic tract; LSTT – Lateral spinothalamic tract; DSTT – Dorsolateral spinothalamic tract; HRP – Horseradish peroxidase; WGA – Wheat germ agglutinin; WGA-HRP – Horseradish peroxidase-conjugated with wheat germ agglutinin; ALQ – Anterolateral quadrant; Po/SG – Posterior nuclei of the caudal-ventral thalamus; PHA-L – *Phaseolus vulgaris* leucoagglutinin; TRITC – Tetramethyl isothiocyanate; FITC – Fluorescein isothiocyanate; SDH – Superficial dorsal horn; DDH – Deep dorsal horn; CTb – Cholera toxin subunit B; VMpo – Posterior part of the ventromedial nucleus; VPL – Ventral posterior lateral; VPI – Ventral posterior inferior; VL – Ventral lateral

Authors	Year	# of Patients	Intervention	Methodology	Key Findings	Complications
**Hyndman et al.** [23]	1943	125	Uni- and bilateral open upper thoracic and cervical cordotomy	Formal Sensory Testing	An anterior cordotomy does not sever central sympathetic connections but does sever central parasympathetic connections, especially those relating to erection and orgasm. Bowel and bladder function can be transiently impaired as well. A cordotomy abolishes visceral pain, shivering, various forms of girdle pain, and itching but not tickle nor deep reflexes	Bladder and bowel dysfunction, motor weakness, reduced sexual function, transient hypotension
**White et al.** [24]	1950	210	Uni - and bilateral open upper thoracic and cervical cordotomy	Long-term formal sensory testing on 20 patients	The study demonstrated that sensations other than pain and temperature were not affected to a significant degree. Furthermore, the degree of analgesia is related to the sensation of a pinprick. Patients with unilateral cordotomy had transient urinary retention, while patients with bilateral cordotomies had long-term issues with urinary retention. Occasional motor weakness was also noted. Late return of	Bladder and bowel dysfunction, Motor weakness, Incisional radicular pain, reduced sexual function, dysesthesia, and reference of pain

					pain is caused by a drop in sensory level, development of hypalgesia, or extension of disease.	
**Nathan et al.** [25]	1951	27	Uni- and bilateral thoracic cordotomy	Histological Correlation for 18 patients	The sensation to micturate and sensation of pain from the bladder lies on the surface of the cord, approximately opposite the dorsal lateral process of the anterior horn in the STT. It lies in the same region as the STT fibers from the sacral segments of the body.	Bladder dysfunction
**Roth et al.**[26]	1952	10	Uni- and bilateral open upper thoracic cordotomy (T1-T2)	Formal Sensory Testing	There is a somatotopic arrangement of pain and temperature fibers with sympathetic (sweat and vasomotor) fibers being widely distributed across the anterolateral quadrant	Anhidrosis
**Johnson et al** [27]	1952	5	Uni- and bilateral open upper thoracic cordotomy (T1-T2)	Formal Sensory Testing	Vasomotor and sweating functions can be affected in a dissociated manner. Autonomic pathways in the spinal cord are widely distributed, and a greater part is not intimately related to the corticospinal tract.	Anhidrosis, transient ptosis and miosis
**White et al** [28]	1956	~300	Uni- and bilateral open upper thoracic cordotomies	Histological correlation of 18 cases	The study determined that in the thoracic cord, sacral fibers were located dorsally just in front of the	Bladder and bowel dysfunction, Motor weakness, incisional radicular pain, burning pain,

					dentate ligament and laterally just deep to the ventral spinocerebellar tract, with thoracic fibers being located ventromedially and lumbar fibers in between. Despite the relative locations, the authors noted that scattered fibers from the leg and buttocks could also be found ventral to the motor roots. Furthermore, they noted that there is a wide variation in the position of axons that conduct pain.	
**Nathan et al. [29]**	1956	15	Unilateral high cervical cordotomy	Formal sensory testing	Reference of pain only occurs by stimuli conveyed through the STT and in the region rendered analgesic. The pain can be referred to the analogous contralateral region or can manifest cranially or caudally. The authors hypothesized that the posterior columns or multisynaptic pain pathways are responsible for this complication.	Reference of pain
**Nathan et al. [30]**	1958	36	Uni- and bilateral thoracic cordotomy	Histological correlation and bladder function testing	Most descending fibers mediating micturition lie in the lateral column on an equatorial plane	Bladder Dysfunction

					passing through the central canal.	
**Osacar et al.** [31]	1961	1	Bilateral upper thoracic cordotomy	Histological correlation	Despite the complete destruction of the STT on the right side and partially on the left side, there was no relief of pain and no detectable loss of cutaneous pain or tactile or temperature sensibility.	-
**Belmusto et al.** [32]	1963	20	Uni- and bilateral open high cervical cordotomy	Pulmonary function testing	Unilateral cervical cordotomies can result in respiratory impairment if pulmonary pathology is present. Bilateral cervical cordotomies in the presence of pulmonary pathology should not be performed. Hypotension and respiratory impairment can be used as a measure of adequate lesioning. Respiratory impairment is caused by damage to descending motor pathways. Low cervical cordotomies do not produce pulmonary dysfunction	Pulmonary dysfunction and hypotension
**Nathan et al.** [66]	1963	8	Unilateral high cervical cordotomy	Histological correlations and electromyography testing in 5 patients	The fibers mediating respiration lie in the anterior part of the lateral column of the cervical cord. These fibers do not descend in the corticospinal	Pulmonary Dysfunction

					tract but rather in the reticulospinal or olivospinal tracts	
**Tenicela et al.**[33]	1968	13 (Retrospective) 41 (Prospective)	Uni- and bilateral high cervical percutaneous cordotomy (C2)	Pulmonary function testing	In a unilateral cordotomy, postoperative respiratory dysfunction is possible with comorbid lung disease. Pulmonary dysfunction is more common in bilateral cordotomies and is related to the total number of afferent neurons interrupted.	Pulmonary dysfunction, transient ataxia and hemiparesis, transient bladder and bowel dysfunction, hypotension, and Horner's syndrome
**Hitchcock et al.** [34]	1967	14	High cervical and thoracic open cordotomy	Pulmonary function testing	The respiratory efferent pathway lies at least 2mm deep to the surface of the spinal cord. Posterior portions of the respiratory tract (i.e., intercostal muscles) are associated with posterior portions of the spinothalamic pathway. Still, the area having a maximal effect on respiration (i.e., diaphragm) is located close to the cervical segments of the STT. Damage to the reticulospinal tract can cause respiratory dysfunction.	Respiratory dysfunction
**Rosomoff et al** [35]	1969	48	Uni- and bilateral high cervical percutaneous cordotomy	Pulmonary function testing	The effects of bilaterality and high analgesia are additive. Respiratory dysfunction is greater after a bilateral	Respiratory Dysfunction

					cordotomy than after a unilateral procedure and greater when a high level of analgesia is achieved compared to a low level. Rather than respiratory motor function, ventilatory control mechanisms are affected.	
**Nathan et al.** [36]	1973	8	Unilateral thoracic cordotomy	Histological Correlation in 6 patients	Following large cordotomy incision and paresis of ipsilateral lower limb, recovery starts within hours. However, if a contralateral large cordotomy is performed, the recovery is removed, and very little re-recovery occurs. Therefore, recovery depends on the contralateral corticospinal tract that must re-cross the spinal cord.	Lower limb paresis/paralysis
**Hitchcock et al** [37]	1974	13	Uni- and bilateral open and percutaneous high cervical cordotomy	Bladder Function Testing	Unilateral cordotomies at the C1 level produce no appreciable change in micturition. Unilateral innervation is enough to retain normal function. Bilateral cordotomies produce sacral analgesia and give rise to micturition disorders. The vesicomotor pathway lies within 3mm of the cord surface in the region	Bladder dysfunction

					of the dentate ligament.	
**Krieger et al.** [38]	1974	10	Bilateral percutaneous cervical cordotomy	Histological Correlations	There are three patterns of respiratory dysfunction: 1) vital capacity is decreased preferentially, 2) CO_2 response is attenuated, and the vital capacity is not affected, and 3) the mixture of the first two patterns. These authors hypothesized that ascending tracts relaying impulses to the respiratory center are disrupted.	Bladder and respiratory dysfunction, hypotension, and hyponatremia
**Moffie et al.** [39]	1975	4	High cervical percutaneous cordotomy	Histological Correlation	The study demonstrated that two patients with lesions located in the posterolateral quadrant showed analgesia, indicating that at the cervical level, nociceptive fibers can be found in the posterolateral quadrant for some patients	Slight paresis, loss of vibratory sense on the left leg, and late onset of new pain
**Noordenbos et al.** [40]	1975	1	Thoracic spinal cord transection with preservation of one anterolateral quadrant	Formal Sensory Testing and Anatomical Correlation	This unique case demonstrated that pain, touch and pressure could be localized on both sides despite the patient only having one anterolateral quadrant.	-
**Nathan et al** [41]	1986	2	Combined commissural myelotomy and unilateral	Formal Sensory Testing	With combined lesions of the dorsal column and STT, there is a complete lack of tactile and	-

			midthoracic cordotomy		pressure sensibility. Lesions to the dorsal column alone cause minimal to slight deficits in tactile and pressure sensibility, while lesions to the STT caused no perceived changes.	
**Polatty et al.** [42]	1986	1	Right unilateral high cervical percutaneous cordotomy (C1-C2)	EEG, polysomnogram, and pulmonary function testing	Patients with pre-existing lung pathology can develop permanent Ondine's curse, which is difficult to manage.	SIADH and orthostatic hypotension
**Lema et al.** [43]	1986	15	Unilateral high cervical percutaneous cordotomy	Pulmonary function testing	After a percutaneous cordotomy, there was a 20% drop in FEV_1.	Respiratory dysfunction
**Nathan et al.** [44]	1990	65	Uni- and bilateral open thoracic cordotomy	Formal sensory testing with histological correlation of 38 patients	The study noted that no form of mechanoreception or tactile sensibility was altered. In addition, graphesthesia, proprioception, and vibratory sense were not affected in 62 of the 65 patients. However, itch was no longer felt, and the cordotomy diminished two-point discrimination.	-
**Lahuerta et al.** [45]	1990	16	High cervical percutaneous cordotomy (C1-C2)	Formal sensory testing	The authors found a highly significant elevation in skinfold pinch thresholds without significant interference in warmth, and hot pain thresholds carried peripherally in	Transient weakness of the ipsilateral leg, non-mirror contralateral pain, and mirror-pain

					unmyelinated fibers. However, a significant difference was observed in the sensation of coolness transmitted by Aδ sensory units. The authors conclude that cordotomy most affects modalities carried by Aδ fibers, including pinprick, cold sensations, and skinfold pinch. They found changes in sensory modalities carried by C fibers, such as warmth and heat pain, but less significantly. Furthermore, they noted that patients did not experience startle, tickle, or erotic sensations when subjected to low-intensity tactile stimulation.	
**Di Piero et al.** [46]	1991	5	Unilateral percutaneous high cervical cordotomy (C1-C2)	Postoperative PET scan	Chronic pain decreases synaptic activity at the thalamic level, either from decreased activity of projecting neurons or attenuated local neuronal firing.	-
**Lahuerta et al.** [47]	1992	12	Uni- and bilateral high cervical percutaneous cordotomy (C1-C2)	Histological Correlations	Respiratory apnea post-cordotomy occurs almost exclusively in patients with coexisting pulmonary pathology. The fibers responsible for respiratory	Respiratory Dysfunction

					dysfunction are intermingled with ascending pain fibers.	
**Nagaro et al.** [48]	1993	66	Uni- and bilateral cervical percutaneous cordotomy	Formal Sensory Testing	There was no correlation between the occurrence of reference of pain (ROPC) and pre-operative pain states or the results of the cordotomy. The authors postulated that ROPC occurs via a subsidiary pathway that is inhibited under normal conditions by feedback inhibition but is released by a cordotomy.	Reference of pain
**Lahuerta et al.** [49]	1994	146	Uni- and bilateral percutaneous cervical cordotomies	Anatomical correlations on 29 patients	Pain fibers at the C2 level are located anterior to the dentate ligament and extend to a line drawn perpendicularly to the medial angle of the ventral horn. However, from the histological diagrams of the lesion provided, it is evident that in 14 of 29 spinal cords, the lesion extended dorsal to the dentate ligament. The best analgesic results were obtained by lesions that extended 5.0 mm deep and destroyed about 20% of the hemicord. There is a somatotopic organization with	Horner's syndrome; Weakness of ipsilateral leg and upper arm; Urinary retention; New pain on the contralateral side; Intensification of pain on the ipsilateral side

					sacral fibers running dorsolaterally and cervical fibers running ventromedially. The authors believed that pain secondary to tissue damage and pinprick is mediated by separate fibers but overlaps in the ALQ.	
**Nathan et al.** [50]	1994	44	Uni- and bilateral ventral and dorsal funiculus lesions	Histological Correlations	An incision in the anterior half can be made without causing motor disturbances. The more posterior the incision reached, the greater the motor deficits. Furthermore, the corticospinal fibers for the upper limb are located more posteriorly in the cranial segments of the spinal cord.	Upper and lower limb motor disturbances
**Friehs et al** [51]	1995	3	Open low cervical or upper thoracic and percutaneous high cervical cordotomies	Formal sensory testing	The study concluded that pain and temperature conduction in the STT are segregated and hypothesized that fibers relating to temperature are located behind the sharp pain fibers of each respective level (e.g., cervical, thoracic, etc)	Mirror-pain syndrome
**Nathan et al.** [52]	2001	63	Uni- and bilateral cervical, thoracic, and lumbar cordotomy	Formal sensory testing and histological correlation	The study concluded that fibers of the STT cross transversely. Lesioning of the STT produced complete analgesia and thermoanesthesia	-

					starting 1-2 segments caudal of the lesion, which they associated with disruption of decussated contralateral STT fibers along with a narrow band of reduced analgesia and thermoanesthesia at the level of the lesion extending 1-2 segments cranially or caudally that they associated with disruption of decussating fibers.	
**Verlato et al.** [53]	2001	18	Unilateral cervical percutaneous cordotomy	Sympathetic function testing	Unilateral percutaneous cordotomies depress some sympathetic indexes derived from heart rate variability, irrespective of side.	-
**Nagaro et al.** [54]	2001	45	Unilateral cervical percutaneous cordotomy (C1-C2)	Formal pain testing	73.3% of patients complained of new pain. An important contributing factor is the loss of pain sensation. The authors hypothesized that disinhibition and direct commissural connections or supraspinal loops might lead to mirror/reference of pain.	Mirror Pain
**Price et al.** [55]	2003	35	Unilateral cervical percutaneous cordotomy (C1-C2)	Pulmonary function testing	A unilateral cordotomy does not worsen respiratory function in patients with pleural	Pulmonary dysfunction

					mesothelioma or other thoracic malignancies.	
**Bowsher et al.** [56]	2005	16	Unilateral cervical percutaneous cordotomy (C2)	Formal sensory testing	Touch, mechanical pain, sharpness, innocuous warmth and cold, and heat pain are dissociable from each other.	-
**Meeuse et al.** [57]	2008	1	High cervical percutaneous cordotomy (C1-C2)	Formal sensory testing at 5-year follow-up with MRI radiographic correlation	The authors noted that the patient had sensory disturbances without motor or autonomic disturbances. Pain and temperature were most affected, but touch and vibration were also abnormal. The authors hypothesized that vibration was affected due to the disruption of second-order fibers of the dorsal columns that synapse at the lateral cervical nucleus.	-
**Gebarski et al.** [58]	2017	1	Unilateral high cervical percutaneous cordotomy (C1-C2)	Postoperative DTI scans	The cordotomy reduced the rostrocaudal directional DTI signal from the lesion in the cervical spinal cord through the pons and mesencephalon. Focal ablation and the accompanying anterograde degeneration contribute to the analgesic effects of cordotomy.	-
**Vedantam et al.** [59]	2017	7	Unilateral high cervical	Postoperative DTI scans	DTI of the spinal cord can detect	-

			percutaneous cordotomy (C1-C2)		microstructural changes with mean diffusivity predicting early postoperative outcomes.	
**Vedantam et al [60]**	2017	10	Unilateral high cervical percutaneous cordotomy (C1-C2)	Postoperative MRI scans	The study found that the cordotomy lesion was hyperintense with a central hypointense foci on T2-weighted MRI images. Furthermore, the lesions were centered in the dorsolateral region of the anterolateral quadrant with the number of pial penetrations corresponding to percentage of total cord area that was lesioned.	-
**Vedantam et al. [61]**	2018	12	Unilateral high cervical percutaneous cordotomy (C1-C2)	Formal sensory testing with radiographic correlation	The study concluded that spinothalamic fibers mediating sharp pain in the arms are located ventromedially to fibers for the legs. In addition, they noted that the fibers mediating sharp pain are distinct from the fibers mediating temperature. They hypothesized that the fibers mediating temperature are located dorsally in a distinct region to fibers for sharp pain, albeit in a similar somatotopic arrangement.	-

**Honey et al.** [62]	2019	20	High cervical percutaneous cordotomy (C1-C2)	Correlation of electrode recording with radiographic placement	The study illustrated that STT fibers from the leg were located dorsolaterally, while fibers from the arm were located ventromedially. However, they found that fibers from the arm can be found more posteromedially that previously noted	-
**Marshall et al.** [63]	2019	19	High cervical percutaneous cordotomy (C1-C2)	Formal sensory testing	The study concluded that there are no C-tactile-lamina-I-spinothalamic projections. But instead, that pleasant touch is mediated by hedonic and discriminative spinal processing.	-
**Vedantam et al.** [64]	2020	10	Unilateral high cervical percutaneous cordotomy (C1-C2)	Postoperative MRI scans	MRI-based radiomic analysis can predict pain outcomes one week after a cordotomy.	-
**Berger et al.** [65]	2021	15	Unilateral percutaneous high cervical cordotomy (C1-C2)	Radiological (MRI) Correlation	A minimal ablation size is needed for analgesia after a cordotomy; however, larger lesions do not indicate better outcomes. MD and FA metrics represent tissue damage and correlate with pain outcomes.	Mirror pain
**Treister et al.** [66]	2022	18	Unilateral percutaneous high cervical cordotomy (C1-C2)	Formal sensory testing	Patients had reduced sensitivity in sensory and pain thresholds on the affected side with increased sensitivity to mechanical	Mirror Pain

					pressure on the unaffected side. Presurgical temporal summation values predicted postoperative mirror pain. The authors hypothesized that mirror pain might be due to central sensitization.	

Supplementary Table 2: Summary of Human Studies

Abbreviations: STT – Spinothalamic tract; DTI – Diffuse tensor imaging; ALQ – Anterolateral quadrant; MD – Mean diffusivity; FA – Fractional anisotropy

Supplementary References

1. Albe-Fessard D, Levante A, Lamour Y. Origin of spino-thalamic tract in monkeys. *Brain Research*. 1974;65(3):503-509. doi:10.1016/0006-8993(74)90238-8

2. Kerr FWL. The ventral spinothalamic tract and other ascending systems of the ventral funiculus of the spinal cord. *Journal of Comparative Neurology*. 1975;159(3):335-355. doi:10.1002/cne.901590304

3. Trevino DL, Carstens E. Confirmation of the location of spinothalamic neurons in the cat and monkey by the retrograde transport of horseradish peroxidase. *Brain Research*. 1975;98(1):177-182. doi:10.1016/0006-8993(75)90518-1

4. Willis WD, Kenshalo DR, Leonard RB. The cells of origin of the primate spinothalamic tract. *Journal of Comparative Neurology*. 1979;188(4):543-573. doi:10.1002/cne.901880404

5. VIERCK CJ, LUCK MM. LOSS AND RECOVERY OF REACTIVITY TO NOXIOUS STIMULI IN MONKEYS WITH PRIMARY SPINOTHALAMIC CORDOTOMIES, FOLLOWED BY SECONDARY AND TERTIARY LESIONS OF OTHER CORD SECTORS. *Brain*. 1979;102(2):233-248. doi:10.1093/brain/102.2.233

6. Levitt M. The bilaterally symmetrical deafferentation syndrome in macaques after bilateral spinal lesions: Evidence for dysesthesias resulting from brain foci and

considerations of spinal pain pathways. *Pain*. 1983;16(2):167-184. doi:10.1016/0304-3959(83)90206-3

7. Greenspan J, Vierck C, Ritz L. Sensitivity to painful and nonpainful electrocutaneous stimuli in monkeys: effects of anterolateral chordotomy. *The Journal of Neuroscience*. 1986;6(2):380-390. doi:10.1523/JNEUROSCI.06-02-00380.1986

8. Vania Apkarian A, Hodge CJ. A dorsolateral spinothalamic tract in macaque monkey. *Pain*. 1989;37(3):323-333. doi:10.1016/0304-3959(89)90198-X

9. Apkarian AV, Hodge CJ. Primate spinothalamic pathways: II. The cells of origin of the dorsolateral and ventral spinothalamic pathways. *The Journal of Comparative Neurology*. 1989;288(3):474-492. doi:10.1002/cne.902880308

10. Vania Apkarian A, Hodge CJ, Apkarian AV, et al. Primate spinothalamic pathways: III. Thalamic terminations of the dorsolateral and ventral spinothalamic pathways. *Pain*. 1989;98(3):323-333. doi:10.1016/0304-3959(89)90198-X

11. Vierck CJ, Greenspan JD, Ritz LA. Long-term changes in purposive and reflexive responses to nociceptive stimulation following anterolateral chordotomy. *Journal of Neuroscience*. 1990;10(7):2077-2095. doi:10.1523/jneurosci.10-07-02077.1990

12. Ralston HJ, Ralston DD. The primate dorsal spinothalamic tract: evidence for a specific termination in the posterior nuclei (Po/SG) of the thalamus. *Pain*. 1992;48(1):107-118. doi:10.1016/0304-3959(92)90137-Z

13. Craig AD. Spinal location of ascending lamina I axons in the macaque monkey. *Journal of Pain*. 2000;1(1):33-45. doi:10.1016/S1526-5900(00)90086-5

14. Weng HR, Lee JI, Lenz FA, et al. Functional plasticity in primate somatosensory thalamus following chronic lesion of the ventral lateral spinal cord. *Neuroscience*. 2000;101(2):393-401. doi:10.1016/S0306-4522(00)00368-7

15. Zhang X, Wenk HN, Honda CN, Giesler GJ. Locations of Spinothalamic Tract Axons in Cervical and Thoracic Spinal Cord White Matter in Monkeys. *Journal of Neurophysiology*. 2000;83(5):2869-2880. doi:10.1152/jn.2000.83.5.2869

16. Andrew D, Craig AD. Spinothalamic lamina I neurons selectively sensitive to histamine: a central neural pathway for itch. *Nature neuroscience*. 2001;4(1):72-77. doi:10.1038/82924

17. Craig AD, Zhang ET, Blomqvist A. Association of spinothalamic lamina I neurons and their ascending axons with calbindin-immunoreactivity in monkey and human. *Pain*. 2002;97(1-2):105-115. doi:10.1016/S0304-3959(02)00009-X

18. Craig AD. Lamina I, but not lamina V, spinothalamic neurons exhibit responses that correspond with burning pain. *Journal of Neurophysiology*. 2004;92(4):2604-2609. doi:10.1152/jn.00385.2004

19. Craig AD (Bud), Zhang ET. Retrograde analyses of spinothalamic projections in the macaque monkey: Input to posterolateral thalamus. *The Journal of Comparative Neurology*. 2006;499(6):953-964. doi:10.1002/cne.21155

20. Craig AD (Bud). Retrograde analyses of spinothalamic projections in the macaque monkey: Input to ventral posterior nuclei. *The Journal of Comparative Neurology*. 2006;499(6):965-978. doi:10.1002/cne.21154

21. Craig AD. Retrograde analyses of spinothalamic projections in the macaque monkey: Input to the ventral lateral nucleus. *Journal of Comparative Neurology*. 2008;508(2):315-328. doi:10.1002/cne.21672

22. Craig AD (Bud). Topographically organized projection to posterior insular cortex from the posterior portion of the ventral medial nucleus in the long-tailed macaque monkey. *Journal of Comparative Neurology*. 2014;522(1):36-63. doi:10.1002/cne.23425

23. HYNDMAN OR, Wolkin J. ANTERIOR CHORDOTOMY. *Archives of Neurology & Psychiatry*. 1943;50(2):129. doi:10.1001/archneurpsyc.1943.02290200029002

24. White JC, Sweet WH, Hawkins R, Nilges RG. Anterolateral cordotomy: Results, complications and causes of failure. *Brain*. 1950;73(3):346-367. doi:10.1093/brain/73.3.346

25. Nathan PW, Smith MC. THE CENTRIPETAL PATHWAY FROM THE BLADDER AND URETHRA WITHIN THE SPINAL CORD. *Journal of Neurology, Neurosurgery & Psychiatry*. 1951;14(4):262-280. doi:10.1136/jnnp.14.4.262

26. Roth GM, Johnson DA, Craig WMcK. Physiologic Effect of Anterolateral Chordotomy in Man. *Journal of Applied Physiology*. 1952;5(6):261-266. doi:10.1152/jappl.1952.5.6.261

27. JOHNSON DA, ROTH GM, CRAIG WM. Autonomic pathways in the spinal cord. *Journal of neurosurgery*. 1952;9(6):599-605. doi:10.3171/jns.1952.9.6.0599

28. White JC, Richardson EP, Sweet WH. Upper thoracic cordotomy for relief of pain; postmortem correlation of spinal incision with analgesic levels in 18 cases. *Annals of surgery*. 1956;144(3):407-420. doi:10.1097/00000658-195609000-00010

29. Nathan PW. REFERENCE OF SENSATION AT THE SPINAL LEVEL. *Journal of Neurology, Neurosurgery & Psychiatry*. 1956;19(2):88-100. doi:10.1136/jnnp.19.2.88

30. Nathan PW, Smith MC. THE CENTRIFUGAL PATHWAY FOR MICTURITION WITHIN THE SPINAL CORD. *Journal of Neurology, Neurosurgery & Psychiatry*. 1958;21(3):177-189. doi:10.1136/jnnp.21.3.177

31. Osácar EM, Meyer AE, Jakab I. A histologically verified bilateral anterolateral chordotomy without cutaneous sensory loss. *Acta Neurochirurgica*. 1961;9(4):525-537. doi:10.1007/BF01809545

32. BELMUSTO L, BROWN E, OWENS G. Clinical Observations on Respiratory and Vasomotor Disturbance As Related To Cervical Cordotomies. *Journal of neurosurgery*. 1963;20:225-232. doi:10.3171/jns.1963.20.3.0225

33. Tenicela R, Rosomoff HL, Feast J, Safar P. Pulmonary Function Following Percutaneous Cervical Cordotomy. *Anesthesiology*. 1968;29(1):7-16. doi:10.1097/00000542-196801000-00006

34. Hitchcock E, Leece B. Somatotopic Representation of the Respiratory Pathways in the Cervical Cord of Man. *Journal of Neurosurgery*. 1967;27(4):320-329. doi:10.3171/jns.1967.27.4.0320

35. Rosomoff HL, Krieger AJ, Kuperman AS. Effects of percutaneous cervical cordotomy on pulmonary function. *Journal of neurosurgery*. 1969;31(6):620-627. doi:10.3171/jns.1969.31.6.0620

36. NATHAN PW, SMITH MC. EFFECTS OF TWO UNILATERAL CORDOTOMIES ON THE MOTILITY OF THE LOWER LIMBS. *Brain*. 1973;96(3):471-494. doi:10.1093/brain/96.3.471

37. Hitchcock E, Newsome D, Salama M. The somatotopic representation of the micturition pathways in the cervical cord of man. *British Journal of Surgery*. 1974;61(5):395-401. doi:10.1002/bjs.1800610518

38. Krieger AJ, Rosomoff HL. Sleep-induced apnea. *Journal of Neurosurgery*. 1974;40(2):168-180. doi:10.3171/jns.1974.40.2.0168

39. Moffie D. Spinothalamic fibres, pain conduction and cordotomy. *Clinical Neurology and Neurosurgery*. 1975;78(4):261-268. doi:10.1016/0303-8467(75)90005-0

40. Noordenbos W, Wall PD. Diverse sensory functions with an almost totally divided spinal cord. A case of spinal cord transection with preservation of part of one anterolateral quadrant. *Pain*. 1976;2(2):185-195. doi:10.1016/0304-3959(76)90114-7

41. Nathan PW, Smith MC, Cook AW. Sensory effects in man of lesions of the posterior columns and of some other afferent pathways. *Brain*. 1986;109(5):1003-1041. doi:10.1093/brain/109.5.1003

42. POLATTY RC, COOPER KR. Respiratory Failure After Percutaneous Cordotomy. *Southern Medical journal*. 1986;79(7):897-899. doi:10.1097/00007611-198607000-00028

43. Lema JA, Hitchcock E. Respiratory Changes after Stereotactic High Cervical Cord Lesions for Pain. *Stereotactic and Functional Neurosurgery*. 1986;49(1-2):62-68. doi:10.1159/000100130

44. Nathan PW. Touch and surgical division of the anterior quadrant of the spinal cord. *Journal of Neurology, Neurosurgery and Psychiatry*. 1990;53(11):935-939. doi:10.1136/jnnp.53.11.935

45. Lahuerta J, Bowsher D, Campbell J, Lipton S. Clinical and instrumental evaluation of sensory function before and after percutaneous anterolateral cordotomy at cervical level in man. *Pain*. 1990;42(1):23-30. doi:10.1016/0304-3959(90)91087-Y

46. Di Piero V, Jones AKP, Iannotti F, et al. Chronic pain: a PET study of the central effects of percutaneous high cervical cordotomy. *Pain*. 1991;46(1):9-12. doi:10.1016/0304-3959(91)90026-T

47. Lahuerta J, Buxton P, Lipton S, Bowsher D. The location and function of respiratory fibres in the second cervical spinal cord segment: Respiratory dysfunction syndrome after cervical cordotomy. *Journal of Neurology, Neurosurgery and Psychiatry*. 1992;55(12):1142-1145. doi:10.1136/jnnp.55.12.1142

48. Nagaro T, Amakawa K, Kimura S, Arai T. Reference of pain following percutaneous cervical cordotomy. *Pain*. 1993;53(2):205-211. doi:10.1016/0304-3959(93)90082-Z

49. Lahuerta J, Bowsher D, Lipton S, Buxton PH. Percutaneous cervical cordotomy: A review of 181 operations on 146 patients with a study on the location of "pain fibers" in the C-2 spinal cord segment of 29 cases. *Journal of Neurosurgery*. 1994;80(6):975-985. doi:10.3171/jns.1994.80.6.0975

50. Nathan PW. Effects on movement of surgical incisions into the human spinal cord. *Brain*. 1994;117(2):337-346. doi:10.1093/brain/117.2.337

51. Friehs GM, Schröttner O, Pendl G. Evidence for segregated pain and temperature conduction within the spinothalamic tract. *Journal of neurosurgery*. 1995;83(1):8-12. doi:10.3171/jns.1995.83.1.0008

52. Nathan PW, Smith M, Deacon P. The crossing of the spinothalamic tract. *Brain*. 2001;124(4):793-803. doi:10.1093/brain/124.4.793

53. Verlato G, Polati E, Speranza G, Finco G, Gottin L, Ischia S. Both right and left cervical cordotomies depress sympathetic indexes derived from heart rate variability

in humans. *Journal of Electrocardiology*. 2001;34(4):309-317. doi:10.1054/jelc.2001.27843

54. Nagaro T, Adachi N, Tabo E, Kimura S, Arai T, Dote K. New pain following cordotomy: Clinical features, mechanisms, and clinical importance. *Journal of Neurosurgery*. 2001;95(3):425-431. doi:10.3171/jns.2001.95.3.0425

55. Price C, Pounder D, Jackson M, Rogers P, Neville E. Respiratory Function After Unilateral Percutaneous Cervical Cordotomy. *Journal of Pain and Symptom Management*. 2003;25(5):459-463. doi:10.1016/S0885-3924(03)00042-3

56. Bowsher D. Representation of Somatosensory Modalities in Pathways Ascending from the Spinal Anterolateral Funiculus to the Thalamus Demonstrated by Lesions in Man. *European Neurology*. 2005;54(1):14-22. doi:10.1159/000086884

57. Meeuse JJ, Vervest ACM, Van Der Hoeven JH, Reyners AKL. Five-year follow-up of a cordotomy. *Pain Research and Management*. 2008;13(6):506-510. doi:10.1155/2008/635197

58. Gebarski SS, Chiravuri S, Foerster BR, Patil PG. Microstructural mechanisms of analgesia in percutaneous cervical cordotomy revealed by diffusion tensor imaging. *Journal of Clinical Neuroscience*. 2017;45:311-314. doi:10.1016/j.jocn.2017.08.031

59. Vedantam A, Hou P, Chi TL, Dougherty PM, Hess KR, Viswanathan A. Use of Spinal Cord Diffusion Tensor Imaging to Quantify Neural Ablation and Evaluate Outcome after Percutaneous Cordotomy for Intractable Cancer Pain. *Stereotactic and Functional Neurosurgery*. 2017;95(1):34-39. doi:10.1159/000453279

60. Vedantam A, Hou P, Chi TL, et al. Postoperative MRI Evaluation of a Radiofrequency Cordotomy Lesion for Intractable Cancer Pain. *AJNR American journal of neuroradiology*. 2017;38(4):835-839. doi:10.3174/ajnr.A5100

61. Vedantam A, Bruera E, Hess KR, Dougherty PM, Viswanathan A. Somatotopy and Organization of Spinothalamic Tracts in the Human Cervical Spinal Cord. *Neurosurgery*. 2019;84(6):E311-E317. doi:10.1093/neuros/nyy330

62. Michael Honey C, Ivanishvili Z, Honey CR, Heran MKS. Somatotopic organization of the human spinothalamic tract: In vivo computed tomography–guided mapping in awake patients undergoing cordotomy. *Journal of Neurosurgery: Spine*. 2019;30(5):722-728. doi:10.3171/2018.11.SPINE18172

63. Marshall AG, Sharma ML, Marley K, Olausson H, McGlone FP. Spinal signalling of c-fiber mediated pleasant touch in humans. *eLife*. 2019;8:1-15. doi:10.7554/eLife.51642

64. Vedantam A, Hassan I, Kotrotsou A, et al. Magnetic Resonance-Based Radiomic Analysis of Radiofrequency Lesion Predicts Outcomes After Percutaneous Cordotomy: A Feasibility Study. *Operative Neurosurgery*. 2020;18(6):721-727. doi:10.1093/ons/opz288

65. Berger A, Artzi M, Aizenstein O, et al. Cervical cordotomy for intractable pain: Do postoperative imaging features correlate with pain outcomes and mirror pain? *American Journal of Neuroradiology*. 2021;42(4):794-800. doi:10.3174/AJNR.A6999

66. Treister R, Honigman L, Berger A, et al. Temporal Summation Predicts De Novo Contralateral Pain After Cordotomy in Patients With Refractory Cancer Pain. *Neurosurgery*. 2022;90(1):59-65. doi:10.1227/NEU.0000000000001734
